# Supplementary material for: A Comprehensive Analysis of Clinical Trials in the COVID-19 Pandemic Era
Source: Medicina (Kaunas). 2020 Jun 26;56(6):315. doi: 10.3390/medicina56060315 (PMC7353878; doi:10.3390/medicina56060315)
Supplement: Supplementary file 1 [file medicina-56-00315-s001.pdf]

Table S1. Summary of characteristics for registered randomized controlled trials.

RD	Country	Masking	Purpose	Primary Outcome	Secondary Outcome	Inclusion Criteria	TN	Treatment	Multicenter	Multicountry	Sponsor
**26 March 2020**	Israel	Blind	SC	SP	C	All COVID-19	30	Anti-inflammatory & Antioxidant oral nutrition vs. iso-caloric nutrition	No	No	Researcher
**27 March 2020**	China	Blind	Treatment	C	None	Pneumonia, CR	90	NK cells vs. IL15-NK cells vs. NKG2D CAR-NK cells vs. ACE2 CAR-NK cells vs. NKG2D-ACE2 CAR-NK cells	No	No	Researcher
**19 March 2020**	USA	Blind	Treatment	C	C	Pneumonia, CR	400	Sarilumab vs. Placebo	No	No	Industry
**11 March 2020**	Spain	OL	Prevention	Diagnosis	Mortality	All COVID-19	3040	Hydroxychloroquine	No	No	Researcher
**26 March 2020**	Israel	OL	Treatment	C	None	Non severe COVID-19	1116	Hydroxychloroquine	No	No	Researcher
**26 March 2020**	USA	OL	Treatment	C	Adverse effects	ARDS, CR	6000	Remdesivir	Yes	Yes	Industry
**18 March 2020**	USA	Blind	Treatment	C	Mortality	Healthy COVID-19 positive Pts	100	PUL-042 Inhalation solution	No	No	Industry
**03 March 2020**	USA	OL	Treatment	Hospital day	Adverse effects	Pneumonia, CR	1600	Remdesivir	Yes	Yes	Industry
**12 March 2020**	China	Blind	Treatment	C	None	ARDS, CR	140	Bevacizumab	Yes	No	Researcher
**18 February 2020**	China	Blind	Treatment	C	C	ARDS, CR	40	Thalidomide, α-interferon, Arbidol, Steroid	Yes	No	Researcher
**25 March 2020**	USA	Blind	Treatment	C	C	Hospitalization with COVID-19	330	Tocilizumab	Yes	No	Industry
**18 February 2020**	China	Blind	Treatment	C	Mortality	Pneumonia, CR	100	Thalidomide	Yes	No	Researcher
**20 March 2020**	Mexico	Blind	Treatment	Mortality	Discharge	Severe COVID-19	500	Hydroxychloroquine	No	No	Industry
**27 March 2020**	Spain	OL	Treatment	Mortality	C	ARDS, CR	200	Steroid	Yes	No	Researcher
**18 February 2020**	China	OL	Treatment	C	C	Pneumonia, CR	400	Steroid	Yes	No	Researcher
**24 March 2020**	USA	Blind	Prevention	Diagnosis	None	Contact with COVD-19	1600	Hydroxychloroquine	No	No	Researcher
**23 March 2020**	Mexico	Blind	Prevention	Diagnosis	Discharge	Contact with COVD-19	400	Hydroxychloroquine	No	No	Industry

**11 March 2020**	Thailand	OL	Treatment	SP	Mortality	Healthy COVID-19 positive Pts	80	Oseltamivir, Hydroxychloroquine vs. Lopipinavir, Ritonavir, Oseltamivir vs. Favipiravir, Lopipinavir, Ritonavir vs. Darunavir, Ritonavir, Oseltamivir, Hydroxychloroquine vs. Favipiravir, Darunavir, Ritonavir, Hydroxychloroquine	No	No	Researcher
**27 March 2020**	Germany	Blind	Treatment	Mental health	Mental health	All	600	Guided online support program	No	No	Researcher
**27 March 2020**	Italy	OL	Treatment	C	C	ARDS, CR	54	Emapalumab vs. Anakinra	No	No	Industry
**13 February 2020**	China	Blind	Treatment	C	CR RNA	ICU admission with COVD-19	120	Anti PD-1 antibody vs. Thymosin	No	No	Researcher
**11 March 2020**	UK	Blind	Prevention	C	C	Healthy COVID-19 positive Pts	40000	Chloroquine or Hydroxychloroquine	No	No	Researcher
**18 February 2020**	China	OL	Treatment	C	C	Pneumonia, CR	60	Bromhexine Hydrochloride, Arbidol, Interferon a 2β vs. Arbidol, interferon a 2β	No	No	Researcher
**07 February 2020**	China	OL	Treatment	C	Mortality	Severe COVID-19	80	IVIG	No	No	Researcher
**05 February 2020**	China	Blind	Treatment	C	C	Pneumonia, CR	400	Arbidol vs. Oseltamivir vs. Lopinavir/ritonavir	No	No	Researcher
**05 February 2020**	China	Blind	Treatment	C	C	Pneumonia, CR	100	Arbidol vs. Arbidol, Interferon atomization	No	No	Researcher
**07 February 2020**	China	Blind	Treatment	Adverse effect	C	Hospitalization with COVID-19	60	Ritonavir, Oseltamivir, ASC9F	No	No	Researcher
**10 February 2020**	China	Blind	Treatment	C	C	Pneumonia, CR	100	Steroid	No	No	Researcher
**25 March 2020**	Hungary	Blind	Prevention	C	C	All	7576	Personailzed health education	No	No	Researcher
**17 March 2020**	China	OL	Treatment	C	C	All COVID-19	150	Favipiravir, Tocilizumab vs. Favipiravir vs. Tocilizumab	Yes	No	Researcher
**25 March 2020**	Norway	OL	Treatment	Mortality	C	ICU admission with COVD-19	700	Remdesivir vs. Hydroxychloroquine	Yes	No	Researcher
**27 March 2020**	Germany	OL	Treatment	SP	Mortality	COVID-19 with ECMO	30	vvECMO	No	No	Researcher
**27 March 2020**	UK	Blind	Treatment	Vaccination	Adverse effects	Healthy adults	510	ChAdOx1 nCoV-19	No	No	Researcher
**25 March 2020**	Denmark	Blind	Treatment	C	Adverse effects	Hospitalization with COVID-19	180	Camostat Mesilate	Yes	No	Researcher

**27 March 2020**	USA	Blind	Treatment	C	SP	Contact with COVID-19	150	Anti-SARS-CoV-2 Plasma vs. SARS-Cov-2 non immune plasma	No	No	Researcher
**17 March 2020**	USA	Blind	Treatment	C	Mortality	Pneumonia, CR	200	Losartan	No	No	Researcher
**20 March 2020**	France	OL	Treatment	C	C	Pneumonia, CR	3100	Remdesivir vs Lopinavir/Ritonavir vs. Interferon β 1a	Yes	No	Researcher
**17 March 2020**	USA	Blind	Treatment	Hospital day	C	Suspected	516	Losartan	No	No	Researcher
**17 February 2020**	Italy	OL	Treatment	SP	C	Pneumonia, CR	380	Arbidol	Yes	No	Researcher
**17 March 2020**	USA	Blind	Treatment	Mortality	SP	ARDS, CR	120	Aviptadil	Yes	Yes	Industry
**12 March 2020**	USA	OL	Treatment	C	Death	Hospitalization with COVID-19	240	NO gas	Yes	No	Researcher
**27 March 2020**	Canada	Blind	Treatment	C	None	All COVID-19	1500	Azithromycin, Chloroquine	No	No	Researcher
**21 February 2020**	USA	OL	Treatment	C	SP	Suspected	440	Remdesivir	Yes	No	Industry
**25 March 2020**	Brazil	OL	Treatment	C	Death	Suspected	440	Hydroxychloroquine + Azithromycin	No	No	Researcher
**25 March 2020**	Canada	Blind	Prevention	SP	Death	Contact with COVID-19	1220	Lopinavir/Ritonavir	No	No	Researcher
**26 March 2020**	Italy	OL	Treatment	C	Death	Pneumonia, CR	100	Colchicine	No	No	Researcher
**18 March 2020**	USA	OL	Prevention	Diagnosis	SP	Medical staff	470	NO gas	No	No	Researcher
**05 March 2020**	Canada	Blind	Prevention	Diagnosis	C	Medical staff	576	N95 Mask	No	No	Researcher
**26 March 2020**	Denmark	Blind	Treatment	Hospital day	C	Hospitalization with COVID-19	226	Azithroomycin vs. Hydroxychloroquine	No	No	Researcher
**27 March 2020**	France	OL	Treatment	Mortality	C	Pneumonia, CR	180	Sarilumab	No	No	Researcher
**03 March 2020**	China	OL	Treatment	Adverse effect	C	Pneumonia, CR	328	Recombinant human interferon $\alpha 1\beta$	Yes	No	Researcher
**10 February 2020**	China	OL	Treatment	Adverse effect	C	Pneumonia, CR	160	ASC09/Ritonavir vs. Lopinavir/Ritonavir	Yes	No	Researcher
**12 March 2020**	USA	Blind	Treatment	SP	C	ICU admission with COVID-19	200	NO gas	Yes	No	Researcher

**26 March 2020**	Denmark	OL	Treatment	Mortality	C	Severe COVID-19	200	Tocilizumab vs. Sarilumab	Yes	No	Researcher
**26 March 2020**	Brazil	OL	Treatment	C	C	Suspected	630	Hydroxychloroquine, Azithromycin	Yes	No	Researcher
**11 February 2020**	China	Blind	Treatment	C	Mortality	ICU admission with COVD-19	140	Vitamin C	No	No	Researcher
**26 March 2020**	Brazil	Blind	Treatment	Mortality	Mortality	ARDS, CR	440	Chloroquine	Yes	No	Researcher
**26 March 2020**	Canada	Blind	Treatment	Mortality	C	High risk of COVID-19	6000	Colchicine	Yes	No	Researcher
**20 March 2020**	China	Blind	Treatment	Number of pts who unable to SCE Tx	SP	All COVID-19	20	Stem Cell Educator-Treated Mononuclear Cells Apheresis	No	No	Industry
**21 February 2020**	China	OL	Treatment	C	CoV-2 RNA	Pneumonia, CR	30	NK Cells	No	No	Researcher
**20 March 2020**	Norway	OL	Treatment	CoV-2 RNA	C	Hospitalization with COVID-19	202	Hydroxychloroquine	No	No	Researcher
**19 February 2020**	Hong Kong	OL	Treatment	C	C	All COVID-19	70	Lopinavir/ritonavir, Ribavirin, Interferon 1 β vs. Lopinavir/Ritonavir	No	No	Researcher
**16 March 2020**	USA	Blind	Treatment	Diagnosis	C	Contact with COVD-19	3000	Hydroxychloroquine	No	No	Researcher

Abbreviations; ARDS, acute respiratory distress syndrome; C, clinical course; CR, COVID-19 related; ECMO, extra corporeal membrane oxygenation; ICU, intensive care unit; IL, interleukin; IVIG, intra venous immune globulin; NK, natural killer; OL, open label; Pts, patients; RD, registration day; SC, supportive care; SP, serum parameter; TN, total number; Tx, treatment; VV, venovenous.

Table S2. Summary of characteristics for non-randomized intentional trials.

RD	Country	Study Method	Allocation	Direction	Purpose	Primary Outcome	Secondary Outcome	TN	Treatment	Multicenter	Multicountry	Sponsor
**25 March 2020**	Italy	Single Group	Single Arm	Prospective	Treatment	Mortality	Clinical course	49	Hyperimmune plasma	No	No	Researcher
**27 March 2020**	China	Single Group	Single Arm	Prospective	Treatment	Clinical course	CoV-2 RNA	4	DAS181	No	No	Researcher
**27 March 2020**	France	Parallel study	Single Arm	Prospective	Treatment	Mortality	Clinical course	584	Naproxen	No	No	Researcher
**20 March 2020**	Italy	Single Group	Single Arm	Prospective	Treatment	Mortality	Laboratory findings	330	Tocilizumab	Yes	No	Researcher
**11 March 2020**	China	Single Group	Single Arm	Prospective	Treatment	Clinical course	Clinical course	10	Sidenafil	No	No	Researcher
**09 March 2020**	China	Single Group	Single Arm	Prospective	Treatment	Adverse event	Mortality	100	Covid-19/aAPC vaccine	Yes	No	Researcher
**26 March 2020**	Italy	Single Group	Single Arm	Prospective	Treatment	Mortality	Laboratory findings	500	Vitamin C	No	No	Researcher
**24 March 2020**	France	Single Group	Single Arm	Prospective	Diagnosis	Clinical course	Laboratory findings	600		Yes	No	Researcher
**19 February 2020**	China	Single Group	Single Arm	Prospective	Treatment	Clinical course	Mortality	100	Lentiviral Minigene vaccine	Yes	No	Researcher
**24 March 2020**	U.S.A.	Single Group	Single Arm	Prospective	Treatment	Clinical course	Mortality	10	Human amniotic fluid	No	No	Researcher
**10 March 2020**	China	Single Group	Single Arm	Prospective	Treatment	Pulmonary image	Pulmonary image	24	Mesenchymal stem cells	No	No	Industry
**19 February 2020**	China	Single Group	Single Arm	Prospective	Treatment	Laboratory findings	Clinical course	20	Bevacizumab	No	No	Researcher
**20 March 2020**	Italy	Single Group	Single Arm	Prospective	Diagnosis	Diagnosis	Laboratory findings	200	VivaDiag™ COVID-19 lgM/IgG Rapid Test	No	No	Researcher
**17 February 2020**	China	Single Group	Single Arm	Prospective	Prevention	Laboratory findings	Mortality	10	Mesenchymal stem cells	No	No	Researcher
**19 March 2020**	Italy	Single Group	Single Arm	Prospective	Treatment	Clinical course	Mortality	30	Tocilizumab	No	No	Researcher
**24 February 2020**	China	Single Group	Single Arm	Prospective	Diagnosis	Diagnosis	Cost effectiveness	230		Yes	No	Researcher
**19 February 2020**	China	Single Group	Single Arm	Prospective	Treatment	CoV-2 RNA	Clinical course	20	Meplazumab	No	No	Researcher

**27 March 2020**	U.S.A.	Single Group	Single Arm	Prospective	Diagnosis	CoV-2 RNA	Clinical course	20	Plasma obtained from patients COVID-19 recovered	No	No	Researcher
**19 February 2020**	China	Single Group	Single Arm	Prospective	Treatment	Adverse event	Clinical course	30	Mesenchymal stem cells	No	No	Researcher
**26 March 2020**	Canada	Parallel study	Non randomized	Prospective	Treatment	Clinical course	Clinical course	1000	Lopinavir/ritonavir vs Hydroxychloroquine vs Barcitinib	Yes	No	Industry
**20 March 2020**	Brazil	Single Group	Non randomized	Prospective	Treatment	Pulmonary image	Mortality	66	Mesenchymal stem cell	No	No	Industry
**21 February 2020**	China	Parallel study	Non randomized	Prospective	Treatment	Pulmonary image	None	30	Fingolimod	No	No	Researcher
**18 March 2020**	China	Parallel study	Non randomized	Prospective	Prevention	Adverse event	Adverse event	108	Vaccine (Adenovirus Type 5 Vector)	No	No	Industry
**11 February 2020**	China	Parallel study	Non randomized	Prospective	Treatment	Clinical course	Clinical course	10	Immunoglobulin of cured patients vs gamma globulin	No	No	Researcher
**24 March 2020**	China	Parallel study	Non randomized	Prospective	Prevention	Diagnosis	Adverse event	2944	Recombinant human interferon α-1b vs. Thymosin α 1	No	No	Researcher
**24 March 2020**	Italy	Parallel study	Non randomized	Prospective	Treatment	Clinical course	Laboratory findings	60	Baricitinib	No	No	Researcher
**26 March 2020**	Italy	Single Group	Non randomized	Prospective	Treatment	Clinical course	Laboratory findings	104	Steroid	No	No	Researcher
**26 March 2020**	Italy	Parallel study	Non randomized	Prospective	Supportive care	Mortality	Clinical course	120	Escinate sodium	Yes	No	Researcher
**20 March 2020**	U.S.A.	Parallel study	Non randomized	Prospective	Prevention	Adverse event	Laboratory findings	45	m-RNA 1273 (vaccine)	No	No	Researcher

Abbreviations; RD, registration day.

Table S3. Summary of characteristics for observational studies.

RD	Country	Study Design	Purpose	Primary Outcome	Secondary Outcome	Inclusion Criteria	TN	Multicenter	Multicountry	Sponsor
**19 February 2020**	China	Prospective	Diagnosis	Diagnosis	None	High risk of COVID-19	450	No	No	Researcher
**24 March 2020**	Italy	Retrospective	Diagnosis	Clinical course	Mortality	Hospitalization with COVID-19	5000	No	No	Researcher
**26 February 2020**	Hong Kong	Retrospective	Supportive care	Mortality	Clinical course	ICU admission with COVID-19	8	Yes	No	Researcher
**23 March 2020**	China	Retrospective	Supportive care	Mortality	Image	Medical staff	275	No	No	Researcher
**06 March 2020**	China	Prospective	Supportive care	Clinical course	Clinical course	Severe COVID-19	120	No	No	Industry
**26 March 2020**	Italy	Retrospective	Treatment	Mortality	Clinical course	Pneumonia, CR	50	No	No	Researcher
**26 March 2020**	Italy	Prospective	Treatment	Mortality	Mortality	COVID-19 who undergo surgery	1000	No	No	Researcher
**17 February 2020**	China	Prospective	Supportive care	Clinical course	Clinical course	All COVID-19	500	No	No	Researcher
**03 March 2020**	China	Retrospective	Treatment	Mortality	Mortality	All COVID-19	201	No	No	Researcher
**27 March 2020**	France	Prospective	Epidemiology	Diagnosis	Clinical course	Who were in China	1000	No	No	Researcher
**25 March 2020**	Germany	Prospective	Treatment	Mortality	Clinical course	ICU admission with COVID-19	4000	No	No	Researcher
**20 March 2020**	Italy	Retrospective	Treatment	Clinical course	Clinical course	Hospitalization with COVID-19	350	No	No	Researcher
**25 March 2020**	USA	Prospective	Treatment	Clinical course	None	None-severe COVID-19	25	No	No	Researcher
**25 February 2020**	China	Retrospective	Treatment	Mortality	None	Suspected	39	No	No	Researcher
**25 March 2020**	France	Prospective	Supportive care	Clinical course	Clinical course	ICU admission with COVID-19	250	No	No	Researcher
**17 March 2020**	China	Retrospective	Diagnosis	Diagnosis	None	Suspected	100	No	No	Researcher

**13 March 2020**	China	Retrospective	Treatment	Clinical course	Clinical course	Pneumonia, CR	120	No	No	Researcher
**27 March 2020**	Israel	Prospective	Diagnosis	Voice analysis	None	Pneumonia, CR	5000	No	No	Researcher
**20 March 2020**	Sweden	Prospective	Supportive care	Kidney function	Clinical course	Suspected	100	No	No	Researcher
**18 March 2020**	China	Prospective	Treatment	Clinical course	Mortality	All COVID-19	30	No	No	Researcher
**27 March 2020**	Italy	Prospective	Treatment	Mortality	Mortality	Suspected	3000	No	No	Researcher
**18 March 2020**	Romania	Prospective	Diagnosis	Image	None	Pneumonia, CR	200	No	No	Researcher
**19 March 2020**	Norway	Prospective	Treatment	Serum parameter	None	Hospitalization with COVID-19	200	No	No	Researcher
**25 March 2020**	USA	Prospective	Treatment	Clinical course	Discharge	ALL	100000	No	No	Researcher
**27 March 2020**	Italy	Prospective	Diagnosis	Prevalence of COVID-19	Prevalence	Continuous immunosuppressive Tx	300	No	No	Industry
**27 March 2020**	Israel	Prospective	Treatment	Clinical course	None	Suspected	1000	No	No	Researcher
**23 March 2020**	UK	Prospective	Diagnosis	Diagnosis	None	Medical staff	400	No	No	Researcher
**05 February 2020**	China	Prospective	Diagnosis	Diagnosis	Mental health	Who were in China	300000	No	No	Researcher
**26 March 2020**	Italy	Prospective	Diagnosis	Image	None	Suspected	100	Yes	No	Researcher
**13 March 2020**	France	Prospective	Epidemiology	Mental health	Mental health	ALL	50000	Yes	No	Researcher
**27 March 2020**	Italy	Prospective	Treatment	Clinical course	Clinical course	Pneumonia, CR	198	No	No	Researcher
**25 March 2020**	Norway	Prospective	Diagnosis	Diagnosis	None	ALL	50000	No	No	Researcher
**24 March 2020**	Italy	Prospective	Diagnosis	Clinical course	None	Pregnant woman with COVID-19	20	No	No	Researcher
**10 March 2020**	China	Retrospective	Diagnosis	Mortality	None	Pneumonia, CR	669	No	No	Researcher
**21 February 2020**	China	Prospective	Diagnosis	Mortality	Clinical course	Neonates with or born by infected mothers	100	Yes	No	Researcher
**27 March 2020**	France	Prospective	Treatment	Mortality	None	Pneumonia, CR	1000	No	No	Researcher

**20 March 2020**	Italy	Prospective	Treatment	Clinical course	None	Pregnant woman with COVID-19	20	No	No	Researcher
**18 February 2020**	China	Prospective	Treatment	Mortality	Clinical course	ICU admission with COVID-19	100	Yes	No	Researcher
**21 February 2020**	China	Prospective	Diagnosis	Diagnosis	Mortality	Suspected	20	No	No	Researcher
**21 February 2020**	China	Prospective	Supportive care	Mortality	Image	Suspected	100	No	No	Researcher
**03 March 2020**	China	Retrospective	Treatment	Mortality	None	All COVID-19	400	No	No	Researcher
**24 March 2020**	Italy	Prospective	Treatment	Clinical course	None	All COVID-19	1000	No	No	Researcher
**29 January 2020**	China	Prospective	Diagnosis	Diagnosis	Prevalence	Suspected	50	No	No	Researcher
**27 March 2020**	France	Prospective	Diagnosis	Diagnosis	Clinical course	COVID-19 with DM	300	No	No	Researcher
**05 February 2020**	China	Prospective	Supportive care	Death	Clinical course	Patient who visit ER	12000	No	No	Researcher
**26 March 2020**	Italy	Prospective	Diagnosis	Serum parameter	Serum parameter	Suspected	110	Yes	No	Researcher
**02 March 2020**	France	Prospective	Diagnosis	Diagnosis	None	Suspected	300	Yes	No	Researcher
**07 February 2020**	France	Prospective	Diagnosis	Diagnosis	Serum parameter	High risk of COVID-19	300	No	No	Researcher
**07 February 2020**	China	Prospective	Supportive care	Mental health	Mental health	ALL	30000	Yes	No	Researcher
**13 March 2020**	Italy	Prospective	Treatment	Real-life data	Mortality	All COVID-19	50	No	No	Researcher
**26 March 2020**	France	Prospective	Diagnosis	Diagnosis	Clinical course	High risk of COVID-19	300	No	No	Researcher
**27 March 2020**	USA	Prospective	Treatment	Clinical course	None	Pregnant woman with COVID-19	1000	No	No	Researcher
**24 March 2020**	Turkey	Prospective	Treatment	Clinical course	None	Healthy adults	200	No	No	Researcher
**24 March 2020**	Spain	Prospective	Diagnosis	Clinical course	Clinical course	Solid organ transplant diagnosed of COVID-19	50	No	No	Researcher
**10 February 2020**	France	Prospective	Treatment	Clinical course	None	Suspected	500	No	No	Researcher

Abbreviations: CR, COVID-19 related; DM, diabetes mellitus; ER, emergency room; ICU, intensive care unit RD, registration day; TN, total number; Tx, treatment.
